# Transcriptomics-based analysis of genes related to lead stress and their expression in the roots of *Pogonatherum crinitum*

**DOI:** 10.3389/fpls.2022.1066329

**Published:** 2022-12-14

**Authors:** Chenlu Zhu, Junbao Yu, Shuyi Cao, Xinyi Wu, Weicai Meng, Xiaolong Hou

**Affiliations:** ^1^ Institute of Forestry and Environment, College of Forestry, Fujian Agriculture and Forestry University, Fuzhou, China; ^2^ Key Laboratory of State Administration of Forestry and Grassland on Soil and Water Conservation of Red Soil Region in Southern China, National Forestry and Grassland Administration, Fuzhou, China; ^3^ Cross-Strait Collaborative Innovation Center of Soil and Water Conservation, Department of Education of Fujian Province, Fuzhou, China

**Keywords:** hyperaccumulator, *Pogonatherum crinitum*, Pb stress, transcriptomics, Pb resistance gene, qRT-PCR

## Abstract

Revealing plants’ tolerance and transport genes to heavy metal stress play an important role in exploring the potential of phytoremediation. Taking the heavy metal lead (Pb) hyperaccumulator plant *Pogonatherum crinitum* (Thunb.) Kunth as the research object, a hydroponic simulation stress experiment was set up to determine the physiological indicators such as antioxidant enzymes and non-enzymatic antioxidants in the roots of *P. crinitum* under different Pb concentrations (0, 300, 500, 1000, 2000 mg·L^-1^). RNA-Seq was performed, the Unigenes obtained by transcriptome sequencing were enriched and annotated by Gene Ontology (GO) and Kyoto Encyclopedia of Genes and Genomes (KEGG) databases, and the differential expression genes (DEGs) of root were screened and verified by quantitative real-time polymerase chain reaction (qRT-PCR). The results are as follows: with the increase of Pb concentration, superoxide dismutase (SOD), catalase (CAT), and ascorbic acid (AsA) content increased. Peroxidase (POD), malondialdehyde (MDA), and ascorbic acid–glutathione (AsA-GSH) cycles showed low promotion with high inhibition. A total of 38.21 Gb of bases were obtained by transcriptome sequencing, and the base quality of each sample reached Q20 and Q30, accounting for 90%, making the sequencing results reliable. Combined with transcriptome sequencing, functional annotation, and qRT-PCR validation results, 17 root Pb-tolerant genes of *P. crinitum* were screened out, which were related to antioxidation, transportation, and transcription functions. Moreover, qRT-PCR verification results under different Pb stress concentrations were consistent with the transcriptome sequencing results and changes in physiological indicators. In brief, the root of *P. crinitum* can adapt to the Pb stress environment by up-regulating the expression of related genes to regulate the physiological characteristics.

## 1 Introduction

Heavy metal pollution is one of the major problems in global soil pollution, posing a threat to the growth of plants, sustainable development, and human health (Huang and Luo, 2022). Therefore, the problem of heavy metal pollution in soil has attracted the attention of many scholars worldwide. Phytoremediation is a soil pollution remediation technology that integrates the advantages of low cost, high efficiency, and large-scale use with a minimal negative impact on soil structure (Jiang et al., 2019). The ability of plants to enrich heavy metals in soil is the key to phytoremediation technology (Ma et al., 2021). Therefore, studying hyperaccumulators and their physiological responses to heavy metal stress has become a hot topic. However, the wild-type hyperaccumulators found in the literature still have shortcomings regarding environmental impact, growth cycle, and response to species to heavy metals (Chen, 2008); therefore, the research is still in the exploratory stage.

*Pogonatherum crinitum* (Thunb.) Kunth is a Pb hyperaccumulator plant with large biomass, which can grow normally under Pb stress with a concentration of upto 20,000 mg·kg^-1^ (Hou et al., 2019). Under Pb stress, *P. crinitum* can improve the total antioxidant capacity by increasing the content of osmotic regulators (Han et al., 2018) and regulating the ascorbic acid–glutathione (AsA-GSH) cycle (Han et al., 2018) to adapt to the Pb stress environment. It shows that *P. crinitum* resists heavy metal stress in various ways; however, the molecular biology strategy of its response to Pb stress is still unclear.

Various studies have shown that plants may respond to heavy metal stress by regulating genes related to antioxidants, transporters, signal transduction, and transcription factors (Auguy et al., 2013). Plants such as *Trigonella foenum-graecum* L. (Alaraidh et al., 2018), *Medicago Sativa* L. (Lou et al., 2018), and *Brassica juncea* L. (Singh et al., 2020) reduce the toxic effects of heavy metals on plants by regulating the expression of antioxidant enzymes and other related genes to varying degrees. Sun et al. (2019) conducted transcriptome sequencing, enrichment analysis, and verification of the Cadmium (Cd) hyperaccumulator plant *Brassica campestris* L. They found that the up-regulation of key genes in the glutathione (GSH) metabolic pathway is crucial in improving the plant’s resistance to Cd stress. *Salvinia minima*, a Pb hyperaccumulator plant, increase the glutamine synthetase SmGS gene’s expression level in plants under stress. The subsequent increase in GSH enzyme activity significantly prevents plants from being poisoned by Pb (Neyi et al., 2012). Another study found that Pb induced the FeABCC1 gene to be significantly expressed in the hyperaccumulator plant *Fagopyrum esculentum* Moench and the transformed yeast showed that tolerance to Pb in plants increased significantly with gene expression and accumulated more Pb (Mizuno et al., 2010).

Rini and Hidayati (2021) found that *Saccharum spontaneum* L. stimulated root growth by up-regulating phytochelatins (PCs) gene to adapt to the stress environment under Pb stress. The hyperaccumulator plant *Sedum alfredii* Hance has a strong ability to enrich a variety of heavy metals. In addition, transporter gene SaPCR2 (Ge et al., 2022) and the member of the heat shock transcription factor gene family SaHsfs (Chen et al., 2018) were overexpressed under Pb stress, and these genes may play a role in the detoxification to Pb. The molecular mechanism of Pb tolerance in the hyperaccumulator plant *P. crinitum* is still unclear. Almost no information about genes and their action mechanisms in response to Pb stress leaves a big gap. Therefore this study aims to investigate the response strategies of *P. crinitum* at the molecular level under different Pb stress levels. According to the research objectives, a hydroponic simulation stress experiment with varying concentrations of Pb was used for this study. This study will provide a basis for further revealing the intrinsic mechanism of plant response to Pb stress.

## 2 Materials and methods

### 2.1 Experimental material and design

The tested *P. crinitum* was cultivated with seeds. The seeds were collected from the lead-zinc mine in Sanming, Fujian Province. The seeds were spread evenly on the seedbed in the greenhouse to cultivate the plants. Watering was done regularly to keep the soil water holding capacity (WHC) at about 70% for the experiment. *P. crinitum* plants of consistent growth were selected, cleaned in the root system, and transplanted into a hydroponic device with a 1/8 Hoagland nutrient solution. Fifteen plants per pot were transplanted. The Pb stress test was initiated after cultivating the plants in the nutrient solution for three days.

Two experiments were set up: one group at 1000 mg·L^-1^ Pb stress treatment (indicated by TS in the text) and a control experiment without Pb stress (indicated by CS in the text). The other group was set up with different concentrations of Pb stress treatment :0, 300, 500, 1000, 2000 mg·L^-1^ (represented by CK, Pb300, Pb500, Pb1000, and Pb2000 in the text). Using the 60 mg·L^-1^ Pb solution prepared by (CH_3_COOH)_2_Pb, the corresponding Pb solution is added to the culture device with the nutrient solution according to the designed stress concentrations. The nutrient solution components are shown in supplementary material; the concentration of KH_2_PO_4_ was reduced from 0.136 g·L^-1^ in the original formula to 0.68 mg·L^-1^ to avoid precipitation (**Table S1**).

After repeated washing with deionized water, the roots of the *P. crinitum* were moved into the stress solution of different Pb concentrations and placed in an artificially controlled incubator for the experiment (conditions were 25°C, 75% humidity, 6000 Lx light intensity, and day and night light time was (16/8) h·d^-1^), and the stress time was 7 d. To ensure that *P. crinitum* can grow normally under hydroponic conditions, hydroponic pots were aerated twice every morning and evening for 10 min and supplemented with the nutrient solution without Pb to the original nutrient solution scale. Three repetitions were used for each treatment.

After the stress experiment, harvested fresh *P. crinitum* plants were washed repeatedly with deionized water and dried adequately with filter paper (It takes about 52 d from seed to harvest fresh *P. crinitum* sample, of which 45 d was required for plants grown from seed to 15 cm and 7 d for hydroponic stress experiment). The fresh root samples were quick-frozen in liquid nitrogen and placed in a -80°C refrigerator for later use. The CS and TS-treated samples were used for high-throughput transcriptome sequencing. The samples treated with CK, Pb300, Pb500, Pb1000 and Pb2000 were used for physiological indicators, RNA extraction and fluorescence quantification. In addition, each treatment was repeated three times.

### 2.2 Experiment methods

#### 2.2.1 Physiological indexes measurement

To prepare the enzymatic solution, 0.2 g of fresh roots were weighed and ground into a homogenate in a mortar with liquid nitrogen; then, put into a 4 mL centrifuge tube; added 1 mL of 0.05 mol·L^-1^ phosphate buffer at pH 7.8, and then fix the volume to 4 mL. The solution was mixed well with a vortexer, then centrifuged in a refrigerated high-speed centrifuge at 4°C with 10,000 rpm for 10 min. The supernatant was taken and put in the 4°C refrigerators for later use. The activities of superoxide dismutase (SOD), catalase (CAT), peroxidase (POD) and malondialdehyde (MDA) were determined by nitrogen blue tetrazolium, ultraviolet absorption, guaiacol, and thiobarbituric acid method, respectively (Zou, 1995). For GSH and ascorbic acid (ASA) determination methods, we used methods mentioned by Ma and Cheng (2003) and Tanaka et al. (1985), respectively. The APX activity was consistent with Nakano and Asada (1981).

#### 2.2.2 RNA extraction, sequencing, and assembly

The root RNA was extracted using the RNA prep Pure polysaccharide and polyphenol plant total RNA extraction kit produced by TIANGEN. The concentration and purity of RNA were detected by ultra-micro spectrophotometer (DeNovix Company, DS-11+Spectrophotometer, the concentration should be greater than 50 ng·μl^-1^). OD values were between 1.8-2.0 to ensure the purity of RNA). 1% agarose gel was prepared; electrophoresis was for about 20 min. The gel was placed on an automatic gel imager to observe the sample’s integrity. After quality inspection of the obtained RNA, a library was established and sequenced using the Illumina Hi Seq 4000 sequencing platform. The resulting clean reads were assembled and evaluated using Trinity (Grabherr et al., 2011). RSEM quantifies the assembled Unigenes.

#### 2.2.3 Functional annotation of Unigenes and differential expression genes analysis

Assembled Unigenes were aligned with non-redundant protein sequence (Nr), a cluster of orthologous groups of proteins (COG), gene ontology (GO), Kyoto encyclopedia of genes and genomes (KEGG), and SwissProt databases and highly similar proteins to annotate the IDs of the Unigenes. The reads count data in the gene expression level were analyzed using DESeq2 to obtain differential genes (Love et al., 2014). Genes with false discovery rate (FDR) < 0.05 and |log2FC| >1 were considered significantly different expression genes. The Blast2go software was used to compare the significantly different Unigenes with the KEGG and GO databases for annotation and enrichment analysis and combined with the functional annotation and pathway enrichment results to screen candidate genes related to Pb resistance.

#### 2.2.4 Reverse transcription and quantitative real-time PCR

The cDNA was obtained by reverse transcription using the Uni All-in-one First-Strand cDNA Synthesis SuperMix for qPCR Reverse Transcription Kit produced by Transgen. The selected primers for Pb-resistant genes were designed using the premier designing tools in National Center for Biotechnology Information (NCBI). Then DNAMAN 8, PCR amplification, and electrophoresis verified the stability of each primer. The primers of the Pb-tolerant gene in the roots of *P. crinitum* are shown in the supplementary materials (**Table S2**). qRT-PCR was performed using the QuanStudio3 system fluorescence quantitative PCR instrument of Fuzhou Dobiotech. The reaction system was: 0.4 μL of upstream and downstream primers (10 μM), 1 μL of cDNA, 10 μL of 2×PerfectStart Green qPCR SuperMix, and Nuclease-free Water 8.2 μL for a total of 20 μL. The reaction process was 94°C for 30 s; 94°C for 5 s; 60°C for 30 s, a total of 40 cycles. Each sample has three biological replicates, and each has three technical replicates, and the relative expression levels of genes were calculated using 2^-ΔΔCt^.

### 2.3 Statistical analysis

Using SPSS 25 software, one-way analysis of variance (One-way ANOVA) and Tukey’s *post-hoc* test were used for multiple comparisons of experimental data and followed by multiple comparisons using the least significant difference (LSD) test. The level of significance was set at P < 0.05 (two-tailed). All the test results are expressed as mean ± standard deviation. The differential gene volcano map, heat map, GO entry enrichment map, and KEGG pathway enrichment map are all drawn using the cloud tools of Omicshare. The dynamic heat map of Omicshare and Adobe Illustrator CC jointly drew the pathway analysis map. The relative expression histogram is drawn using Origin 2017.

## 3 Results

### 3.1 Physiological indexes under different Pb stress

Data in **Table 1** show that, under Pb stress, SOD, CAT, and ASA content showed an upward trend with the increase of Pb concentration, and the differences among the treatments were greater than those of CK (*P* < 0.05). SOD and CAT treated with Pb2000 increased by 24.53% and 66.34%, respectively, compared with CK. The contents of POD, aseorbate peroxidase (APX), and GSH in roots reached were higher when treated with Pb500 and increased by 14.68%, 444.22%, and 30.37%, respectively, compared with the control. When the concentration of Pb was less than 1000 mg·kg^-1^, the MDA content increased with the concentration increase. The effects of different Pb stress on these indicators are low promotion and high inhibition.

**Table 1:** The physiological characteristics in the roots of *P. crinitum* under different Pb stress concentrations.

	Treatment				
Physiological indicators	CK	Pb300	Pb500	Pb1000	Pb2000
SOD activity	1832.42 ± 46.11d	2170.04 ± 56.84b	2367.1 ± 82.14a	1971.36 ± 123.14c	2281.86 ± 62.79ab
POD activity	341.72 ± 3.90c	367.70 ± 18.76b	391.88 ± 3.26a	369.74 ± 11.85b	374.20 ± 8.19b
CAT activity	34.69 ± 2.05c	40.05 ± 3.21c	48.78 ± 4.07b	51.38 ± 3.52ab	57.71 ± 2.95a
MDA content	10.63 ± 1.47c	11.57 ± 1.55bc	13.81 ± 2.21b	26.13 ± 5.11a	11.4 ± 1.67bc
APX activity	0.09 ± 0.01b	0.1 ± 0.01b	0.48 ± 0.1a	0.17 ± 0.04b	0.14 ± 0.03b
ASA content	5.02 ± 0.49c	6.13 ± 0.40b	5.03 ± 0.19c	5.44 ± 0.26c	6.69 ± 0.65a
GSH content	1.22 ± 0.04d	1.36 ± 0.06b	1.59 ± 0.04a	1.27 ± 0.04cd	1.29 ± 0.04c

Different lowercase letters in the same row represent significant differences among different treatments (P < 0.05).

### 3.2 Transcriptome sequencing analysis under Pb stress

#### 3.2.1 Sequencing results

Two groups of samples were assembled and filtered to obtain a total of 38.21 Gb of total bases, the GC ratio was 52.71%-53.14%, and the base composition was balanced. The number of bases with base quality values above Q20 in each sample account for more than 95%, and those with base quality above Q30 were more than 90%, indicating that each sample’s sequencing quality is high and meets the requirements for library construction (**Table S3**).

#### 3.2.2 Analysis of DEGs

**Figure 1:**
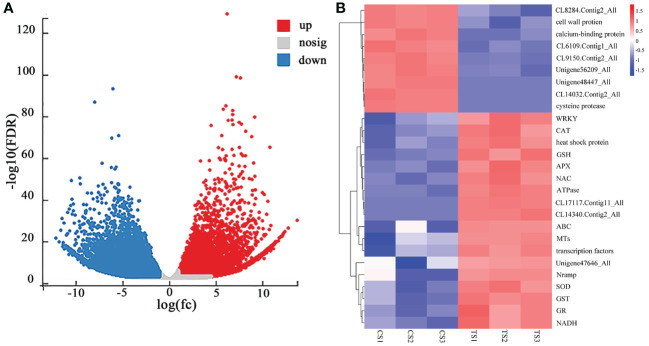
**(A)** CS-vs-TS difference volcano map of roots, and **(B)** heat map of the expression of some Pb-tolerant DEGs in roots of *P. crinitum.* (1000 mg·L^-1^ Pb stress treatment is indicated by TS and control experiment without Pb stress is indicated by CS).

Based on the different analysis results, genes with FDR < 0.05 and |log2FC| >1 were screened as significantly different genes (**Figure 1A**). There were 51,281 unigenes with significant differences (*P* < 0.05), of which 21,138 were up-regulated (accounting for 41.22%), and 30,143 were down-regulated. **Figure 1B** shows that the genes related to antioxidant enzymes were up-regulated, consistent with the response trend of physiological indicators in roots under Pb stress. In addition, the expression levels of genes related to heavy metal transport [ATP-binding cassette (ABC), natural resistance-associated macrophage proteins (Nramp), metallothioneins (MTs)], transcription factor genes (WRKY, NAC), ATPase, GTP-binding proteins, heat shock proteins, and disease course-related proteins (Unigene47646_All) were increased. However, genes related to cell wall proteins (cell wall protein, *CL9150.Contig2_All*, *Unigene48447_All*) and genes related to plant hydrolysis and metabolism (caspases, calcium-binding proteins, *CL14032.Contig2_All*, *Unigene56209_All*, *CL6109.Contig1_All*) showed down-regulated expression. This may be because Pb stress damages the cell wall and some mechanisms related to plant growth and metabolism, hindering its expression.

#### 3.2.3 GO enrichment analysis of DEGs

**Figure 2:**
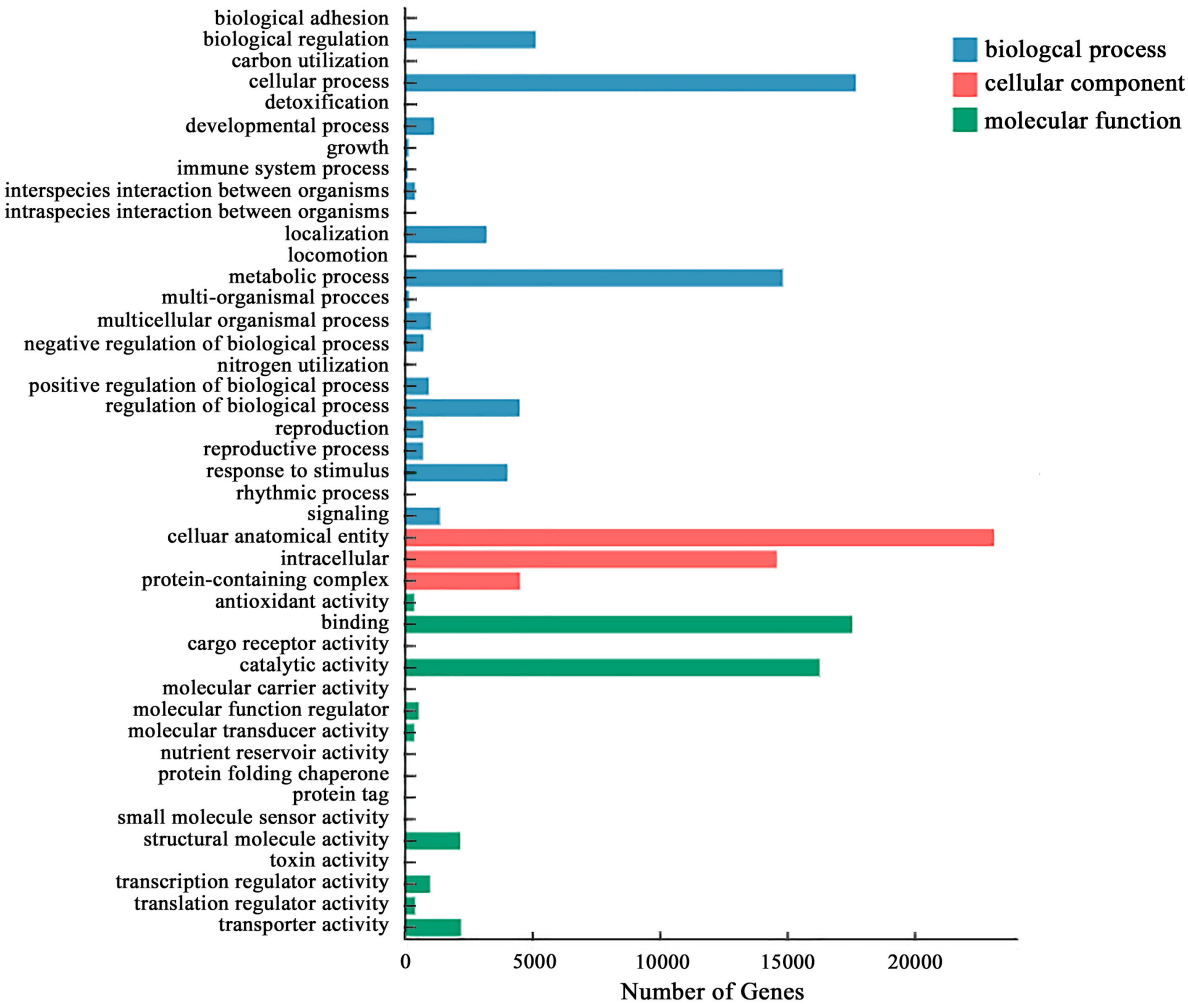
Gene ontology (GO) enrichment classification map of differentially expressed genes in the roots of *P. crinitum* under Pb Stress.

By comparing with the GO database, the differential genes in the roots of *P. crinitum* under Pb treatment are annotated to 44 GO terms, divided into three categories: biological process, cell component, and molecular function. The main functional items in the biological process were the metabolic process and regulation. In the molecular function, the entries with the most significant number of DEGs were binding, catalytic activity, and transporter activity. In the cellular component, the most enriched number was the cellular anatomical entity. It indicates that the root system of *P. crinitum* under Pb stress might adapt to the Pb stress environment through metabolism, generation of new substances, catalytic enzyme activity, and transport of heavy metals (**Figure 2**).

As shown in **Table S4**, a total of 8447 DEGs were significantly enriched in 42 secondary entries related to Pb resistance, including antioxidant enzymes, transport, transcription, ubiquitin, and signaling (*P* < 0.05). The complete details are shown in (**Table S4**). Among them, 1773 DEGs and 1677 DEGs were enriched in antioxidant-related and transcription-related GO items, respectively. Among the significantly enriched entries, four items are related to transport, accounting for 9.53%. The GO items related to signaling account for 6.12%, the DEGs enriched in the GO items related to protein kinases were the most, and the genes significantly enriched in the GO term “protein kinase activity” account for 2.13%.

#### 3.2.4 KEGG enrichment analysis of DEGs

**Figure 3:**
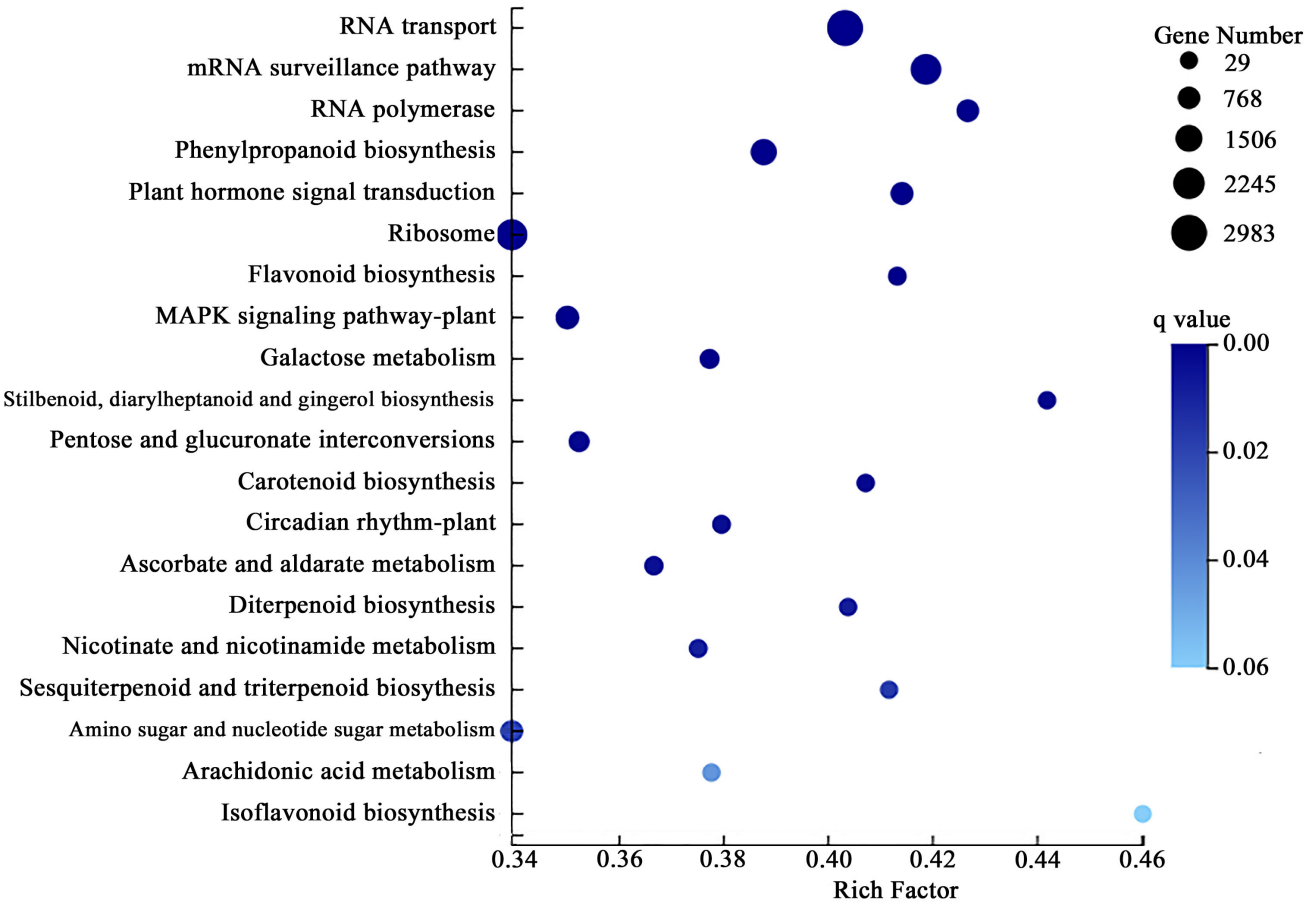
Kyoto encyclopedia of genes and genomes (KEGG) enrichment analysis of differential expression genes (DEGs) in the roots of *P. crinitum* under Pb Stress.

Annotation and enrichment analysis are carried out by comparing the DEGs in the KEGG pathway database’s root system. A total of 15,291 differential genes were significantly enriched in 25 pathways (*P* < 0.05). DEGs on the “RNA transport” pathway were the most significant and enriched, with a total of 2983, accounting for 19.51% of the total DEGs significantly enriched in the KEGG database (**Figure 3**).

The root differential genes were mainly enriched in “Phenylpropanoid biosynthesis,” “MAPK signaling pathway - plant,” and “Plant hormone signal transduction,” accounting for 8.96%, 6.29%, and 5.48%, respectively. Among the KEGG pathways significantly enriched in DEGs, “Flavonoid biosynthesis,” “Ascorbate and aldarate metabolism”, “Isoflavonoid biosynthesis,” “Plant-pathogen interaction” and “Cutin, suberine, and wax biosynthesis,” were all related to plant resistance to heavy metals related, accounting for 49.87% of the total DEGs significantly enriched in the KEGG database. These results indicated that the root system of *P. crinitum* may respond to Pb stress mainly through the transport system. At the same time, signal transmission, antioxidant enzyme, and disease resistance systems also respond to Pb stress. Moreover, KEGG pathways such as “Flavone and flavonol biosynthesis,” “Alanine, aspartate and glutamate metabolism,” “Phenylalanine metabolism,” “Glutathione metabolism,” “ABC transporters,” “Basal transcription factors,” and “Peroxisome” are all related to Pb resistance. A total of 2507 DEGs were enriched, accounting for 7.06% of all differential genes enriched in the KEGG database.

### 3.3 Pathway analysis of Pb-tolerant candidate genes in roots of *P. crinitum*

Combined with the expression and functional annotation results of differential genes, DEGs with significant differences in Pb tolerance were screened from the GO items and significantly enriched KEGG pathways. The genes related to root antioxidants were: *CL17117.Contig11*, *CL17117.Contig17* and *CL762.Contig2* was all involved in the “Peroxisome” pathway, which belongs to the peroxisome targeting signal (PTS1 type) in the antioxidant enzyme system.

**Figure 4:**
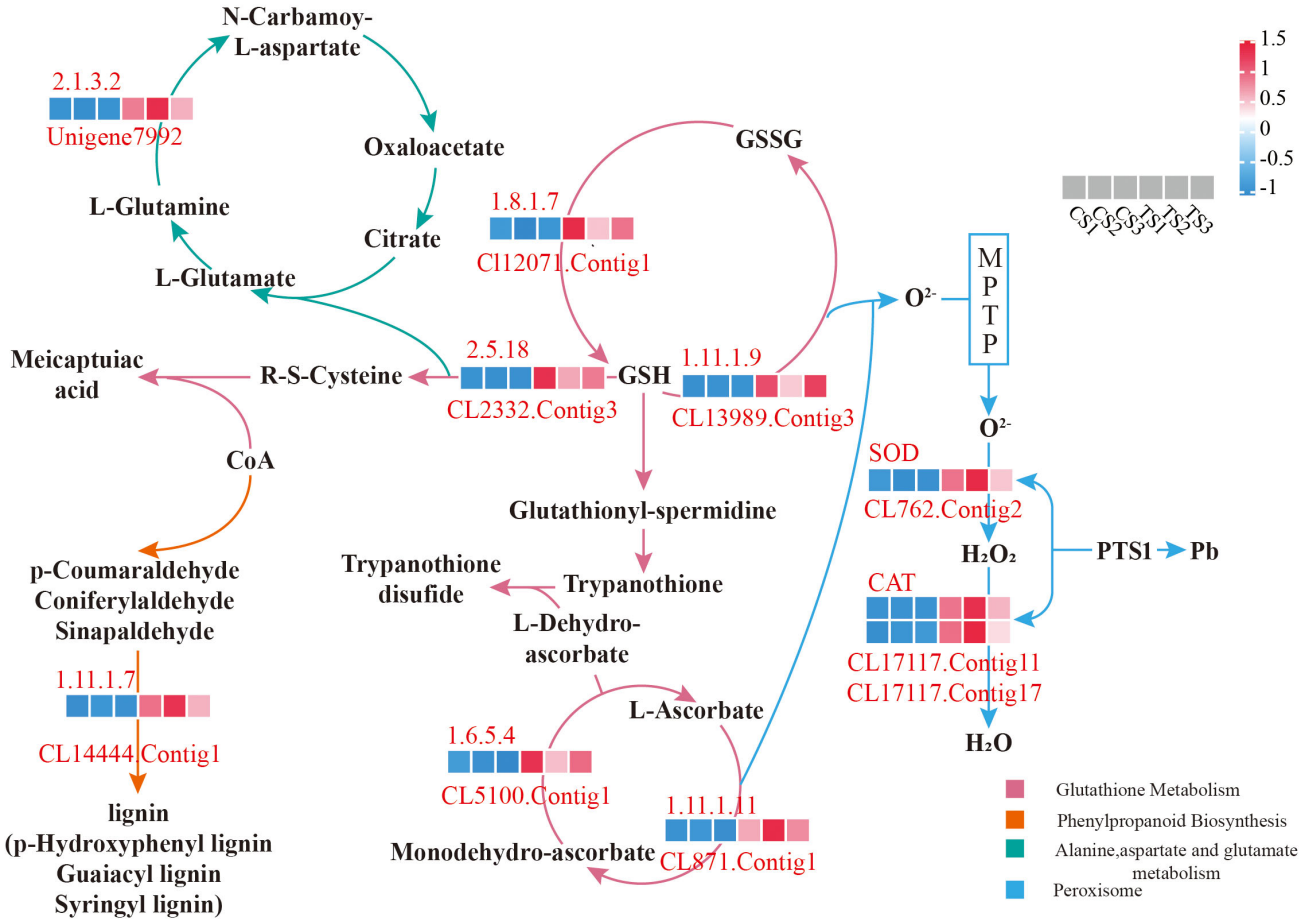
Partial possible molecular mechanism of Pb tolerance in roots of *P. crinitum*.

As shown in **Figure 4**, there were 5 DEGs associated with the ASA-GSH cycle:and all acted on Glutathione metabolism. Among them, *CL871.Contig1* and *CL5100.Contig1* were located at “1.11.1.11” and “1.6.5.4”, respectively, and both sides were L-Ascorbate and Monodehydro-ascorbate, but the two work in the opposite direction. It indicates that these two genes acted on the redox of ascorbic acid in the roots of *P. crinitum*. *CL12071.Contig1* was located at “1.8.1.7” and acts on the transition from GSSG to GSH, while *CL13989.Contig3* was located at “1.11.1.9” and acts on GSH to GSSG. *CL2332.Contig13* was located in “2.5.1.18”, and the back end of this gene is meicaptuiac acid, indicating that this gene may be involved in converting glutathione to meicaptuiac acid, thereby adapting to the Pb stress environment.

A total of 4 root-Pb tolerance genes related to transport were screened: *CL1174.Contig14* participates in “ABC transporters” and is located as an ABC-B class transporter, *CL4795.Contig9*, *CL12532.Contig3* and *CL14340.Contig2* were annotated as a natural resistance-associated macrophage protein, mitochondrial import inner membrane translocase subunit, and COPII protein in the “Protein processing in endoplasmic reticulum” pathway, respectively, indicating that roots under Pb stress might alleviate the toxicity through the differential expression of these transporter-related genes.

Furthermore, five root DEGs were also screened to be related to plant signal transduction and transcription under Pb stress: *Unigene46823* was involved in “Plant-pathogen interaction”, located in “WRKY”, and played a role in the induction of defense-related genes to resist abiotic stress. *CL8121.Contig3* was involved in the “MAPK signaling pathway - plant,” a copper ion exporting ATP enzyme, and acted on the defense response pathway of the plant hormone Ethylene. *CL14444.Contig1* was involved in “1.11.1.7” in “Phenylpropanoid biosynthesis” in **Figure 4** as the activation gene of heat shock protein ATPase, and the upstream and downstream were aldehyde and lignin, respectively, indicating that this gene might be involved in the conversion of aldehydes into lignin, thus responding to the Pb stress environment. *CL1583.Contig2* was annotated as a two-component system protein gene of the “Plant hormone signal transduction” pathway. As shown in **Figure 4**, *Unigene7992* participates in the “Alanine, aspartate and glutamate metabolism” pathway, which was located in the process of “2.1.3.2” acting on the conversion of L-Glutamine to L-Aspartate, and the pathway belonged to the conversion of L-Glutamate.

### 3.4 qRT-PCR validation and analysis of Pb-tolerant candidate genes

**Figure 5:**
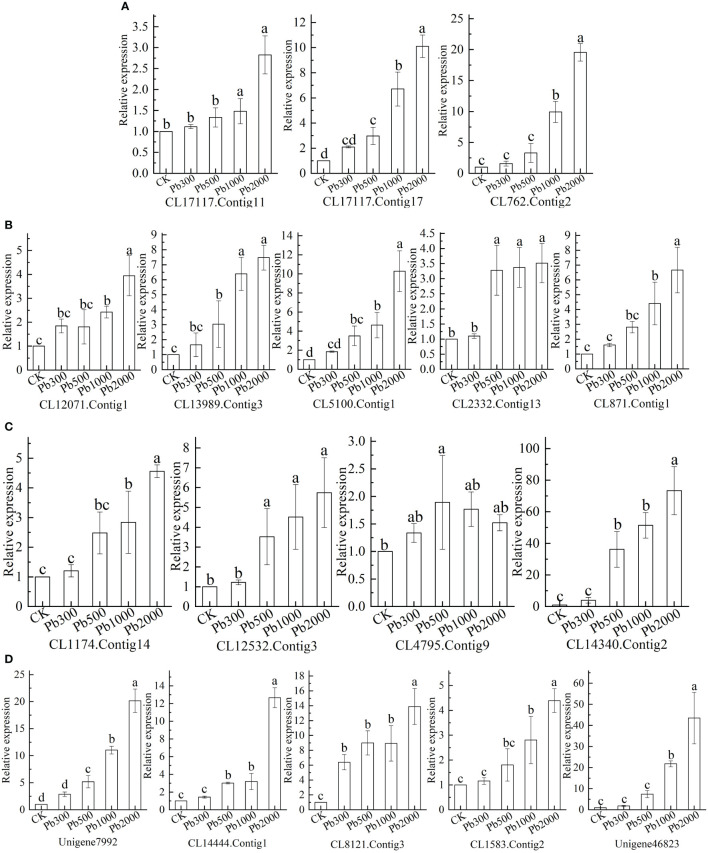
The expression of **(A)** antioxidant-related genes, **(B)** Ascorbic acid–glutathione (ASA-GSH) cycle-related genes, **(C)** transporter-related genes, and **(D)** signal transduction and transcription-related genes in the roots of *P. crinitum* under different lead stress concentrations.

The expression levels of the selected DEGs were analyzed to verify the transcriptome sequencing results and Pb-tolerant candidate genes in roots. As shown in **Figure 5A**, the relative expression of *CL17117.Contig17* increases with the increase of Pb concentration, and the difference between treatments was significant (*P* < 0.05). Under the treatments of different Pb concentrations (**Figure 5D**), the relative expression levels of *CL8121.Contig3* were significantly higher than those in control (*P* < 0.05), and the expression under Pb2000 treatment was significantly higher than that in other treatments (*P* < 0.05), compared with Pb1000 treatment, the up-regulation degree was increased by 55.52%. In addition to Pb300, the relative expression levels of *CL13989.Contig3*, *CL5100.Contig1*, *CL2332.Contig13* (**Figure 5B**), *CL12532.Contig3*, *CL14340.Contig2* (**Figure 5C**) and *Unigene7992*, *CL14444.Contig1* (**Figure 5D**) under each treatment was significantly greater than those of CK (*P*<0.05). The gene expression under the Pb2000 treatment was 7.47, 10.28, 3.52, 5.75, 73.36, 20.16, and 12.65 times that of the control.

The relative expression levels of *CL17117.Contig11*, *CL762.Contig2* (**Figure 5A**), *CL871.Contig1*, *CL12071.Contig1* (**Figure 5B**), *CL1583.Contig2* and *Unigene46823* were significantly higher than CK under Pb1000 and Pb2000 treatments (*P* < 0.05). The expression levels of these genes under Pb1000 treatment are increased by 48.21%, 892.11%, 341.28%, 142.37%, 183.97%, 180.51% and 207.94% compared with CK, respectively. Comparing Pb1000 with Pb2000 treatment, the expression levels were increased by 90.72%, 97.05%, 51.02%, 62.67%, 60.57%, 56.61% and 99.55%, respectively. The relative expression of *CL4795.Contig9* at the Pb concentration of 1000 mg·L^-1^ was significantly higher than that of CK (*P* < 0.05), which is 1.52 times that of the control (**Figure 5C**).

## 4 Discussion and conclusions

The main way for plants to deal with Pb toxicity is to improve their antioxidant capacity (Song et al., 2015). In this study, with the increase of Pb concentration, the activities of POD, SOD and CAT enzymes in the roots of *P. crinitum* generally showed an upward trend. This is because Pb stress activates antioxidant enzymes in the roots, and by increasing their activity, it can remove excess reactive oxygen species (ROS) in the plant and improve the antioxidant capacity of plants to improve the Pb tolerance. *Iris ensata* (Bo et al., 2021), *Brassica juncea* (Małecka et al., 2019), *Medicago sativa* (Helaoui et al., 2020) species have similar response mechanisms. In this study, the content of MDA in roots of *P. crinitum* increased with the increase of Pb stress, indicating that Pb stress caused damage to root membrane lipidation and stimulated the antioxidant system. At Pb2000, the MDA content was significantly lower than other treatments, which is because the antioxidant enzymes in the plants scavenge excess ROS, and the antioxidant defense system alleviates Pb toxicity (Cedeno and Swader, 1972; Mao et al., 2019).

In this study, the GSH content and APX enzyme activity in the roots of *P. crinitum* showed an increasing trend with the concentration increase. This is because the reactive oxygen system was out of balance under Pb stress, and GSH and ASA undergo a reduction reaction with ROS, thereby maintaining dynamic balance (Wang et al., 2021). APX participates in the ASA-GSH cycle as a coenzyme and acts together with POD and CAT to decompose H_2_O_2_ (Qin et al., 2018). When the concentration is more significant than 500 mg·L^-1^, the non-enzymatic antioxidants in *P. crinitum* decreased with the concentration increase. This may be because the high concentration of Pb inhibits the enzymatic reaction in the GSH-ASA cycle, and the synthesis of GSH and ASA is hindered (Liu et al., 2019).

In this study, Pb-resistant candidate genes were screened by transcription sequencing of the roots of TS and CS. GO enrichment analysis found that the most significant GO entry for root DEGs was the metabolic process, followed by a catalytic activity. One of the main ways in which plants respond to external stimuli is metabolite regulation, which can resist abiotic stress by metabolizing or generating secondary metabolites (Sarvajeet and Narendra, 2010). The DEGs in the root system of *P. crinitum* were significantly enriched in GO items because plants produce a large amount of ROS under abiotic stress, and scavenging excess ROS is the main way for plants to adapt to stress and toxicity (Lu et al., 2020). In addition, root DEGs are also significantly enriched on nicotinamide adenine dinucleotide phosphate (NADPH)-related terms. Under Pb stress, antioxidant-related genes in roots promote nicotinamide adenine dinucleotide (NAD+) generation to ensure the reduction reaction of NADPH and inhibit the generation of reactive oxygen species (Zhang et al., 2014). In addition to antioxidant-related GO terms, root DEGs were significantly enriched in functional terms related to transcription, transport, and protein kinases. The possible reason is that the root system is an organ with a high degree of Pb enrichment. Under stress, many gene-regulated plants can improve stress resistance in different ways, which is consistent with Yuan’s research results (Yuan et al., 2017). DEGs in roots of *P. crinitum* were significantly enriched in Pb tolerance-related pathways such as “Phenylalanine metabolism” and “Phenylpropane biosynthesis” under Pb stress. This is because phenolic compounds such as flavonoids and lignin, as the products of phenylpropane metabolism, mainly play a role in removing excess ROS in plants and improving plant antioxidants (Xie and Zhang, 2003), and phenylalanine is involved in this metabolic process as the main ammonia enzyme (Ouyang and Xue, 1988); therefore, the roots of *P. crinitum* can promote phenylpropane biosynthesis through gene regulation and improve its tolerance to Pb stress, which is consistent with the response of the phenylalaninase gene of *Oryza satival* L. subsp. indica to heavy metal stress (Huang et al., 2012). In addition to antioxidant and transport-related KEGG pathways, root differential genes were significantly enriched in signal transduction-related pathways such as “MAPK signaling pathway-plant” and “Plant hormone signal transduction”. This is because signal transduction is activated by the inducement of an abiotic stress environment, in which ROS and plant hormones generated by stress are essential components in the signal transduction process (Wang et al., 2012). Besides, DEGs are also significantly enriched in pathways such as “Flavonoid biosynthesis” and “Ascorbic acid and aldehyde salt metabolism”.

In this study, *CL17117.Contig11*, *CL17117.Contig17* and *CL762.Contig2* in the “peroxisome” pathway were up-regulated expressed, the peroxisomal targeting signal 1 (PTS1) in the antioxidant system. As an important organelle in response to abiotic stress, peroxisomes cannot generate related enzymes by themselves, and PTS is required to direct enzyme precursors into peroxisomes (Zhao, 2000). Pb stress may stimulate the production of PTS1 by peroxisomes in the roots of *P. crinitum*, promotes the directional transport of ribosomes and the expression of these genes to synthesize antioxidant enzymes such as CAT and SOD, and scavenge O^2-^. Under Pb stress, *CL14444.Contig1* is up-regulated in the “phenylpropane biosynthesis” pathway as an upstream gene in the lignin synthesis pathway, which is because this gene promotes the conversion of aldehydes to lignin. As an important component of the cell wall, the increase of lignin content means that the root system can improve the stress resistance of plants by inhibiting the production of oxygen free radicals (Ortega and Peragón, 2009) and increasing the strength of the cell wall (Long et al., 2021). This result is consistent with the response strategy of *Pyrus pyrifolia* to abiotic stress (Lu et al., 2015).

In this study, with the increase of Pb stress concentration, the relative expression levels of CAT, SOD, and POD-related genes gradually increase, which is consistent with the changing trend of root antioxidant enzyme activities, indicating that *P. crinitum* may adjust the enzyme activity of the plant through these antioxidant-related genes to adapt it to the Pb stress environment. Studies have shown that under heavy metal stress, antioxidant-related genes in *Vicia faba* L. (Liu et al., 2020), *Triticum aestivum* L. (Navabpour et al., 2020), and *Phytolacca americana* L. (Zhao et al., 2012) plants are also up-regulated and their enzyme activity changes. With the increase of Pb stress concentration, the relative expression levels of the GSH-ASA cycle-related genes are all up-regulated, and all are consistent with the non-enzymatic antioxidant activity. *CL12071.Contig1* and *CL13989.Contig3* are annotated as glutathione reductase (GR) and glutathione peroxidase (GSH-Px), respectively. The up-regulated expression of the two may be because Pb stress induces GR gene expression and promotes the expression of reduced GSSG to GSH. GSH regulates intracellular and extracellular osmotic pressure and chelates with heavy metals (Sousa et al., 2015), reducing Pb toxicity. The expression of GSH-Px further promotes the reaction between GSH and reactive oxygen species to generate glutathione oxidized (GSSG) (Zhao, 2017). Therefore, the GSSG-GSH cycle maintains plants’ antioxidant capacity and further improves plants’ stress resistance. *CL871.Contig1* and *CL5100.Contig1* were up-regulated with increasing concentration and annotated as APX, and monodehydroascorbate reductase (M-DHAR), respectively. Under Pb stress, *P. crinitum* produced excess ROS and reacted with ASA. The up-regulated expression of the APX gene increases the activity of this enzyme and promotes the oxidation of ASA (Zhou and Mo, 2003). The product of this reaction, monodehydroascorbate (M-DHA) is unstable, and the stress environment stimulates the upregulation of dehydroascorbate reductase (DHAR) gene, which accelerates the reduction of reductase to ASA, thereby further accelerating the scavenging of reactive oxygen species (Li et al., 2007). The oxidation product of M-DHA is dehydroascorbate (DHA), which participates in the metabolic process of spermidine, which can improve the activity of plant antioxidant enzymes and maintain the balance of reactive oxygen species (Liu et al., 2007). *CL2332.Contig13* is expressed on the pathway of GSH conversion to cysteine, which is consistent with the response mechanism of *Porphyra yezoensis* Ueda under Pb stress (Zhou et al., 2011); it is because cysteine cannot only directly react with heavy metals but also reduce the toxicity of heavy metals to plants using transporters (Zhao et al., 2012; Zhang et al., 2017; Abdulla et al., 2019).

The natural resistant macrophage protein gene *CL4795.Contig9* was also up-regulated by Pb stress induction. It is because the protein is tissue-specific and plays a decisive role in the uptake of Pb in the root system of *P. crinitum* (Liu et al., 2022). The root *CL14340.Contig2* belongs to the coat protein II (COP II) in the endoplasmic reticulum, and its expression is up-regulated under Pb stress, which promotes the transport of COP II vesicles to proteins which respond to Pb stress (Zhang et al., 2020). Genes related to transcription factors such as WRKY, v-myb avian myeloblastosis viral oncogene homolog (MYB), and ZRT, IRT-like protein (ZIP) were highly expressed under Pb stress. The possible reason is Pb stimulates the expression of these transcriptomic factors through signal transduction and responds positively to stress, thereby inducing the synthesis of enzymes related to defense against stress and further improving plant stress resistance (Yang et al., 2020). *CL8121.Contig3* acts on the ethylene response pathway, and Pb stress stimulates the expression of this gene, thereby promoting plant ethylene response to the stress environment. Ethylene metabolism can promote the synthesis of antioxidant enzymes and cell lignification to improve plant cell defense capacity (Lu et al., 2020). The two-component system is one of the most important systems in plant hormone signaling, *CL1583.Contig2* regulates the regulator in this system, and Pb stress stimulates the expression of this gene, transmits the signal received by the sensor, and induces the expression of tolerance genes (Lei et al., 2004). Pb stress induces up-regulated expression of *Unigene7992* in the “Alanine, aspartate and glutamate metabolism” pathway, which may be because the gene promotes the synthesis of aspartate and interacts with other proteins to participate in the stress response (Wu et al., 2013). At the same time, its subsequent products, oxaloacetic acid, and citric acid, not only improve the activity of antioxidant enzymes (Basit et al., 2021) but also react with the product glutamic acid and heavy metals, reducing the effectiveness of Pb elements in plants (Guo et al., 2017).

## Data availability statement

The original contributions presented in the study are publicly available. This data can be found here: https://www.ncbi.nlm.nih.gov/sra/PRJNA887529.

## Author contributions

CZ performed the experiments, carried out most data analysis, and wrote the manuscript. JY, SC, XW, WM and XH advised on the experiments and assisted with experiments. XH guided the entire experiment and corrected the manuscript. All authors contributed to the article and approved the submitted version.
