# Supplementary material for: Transcriptomics-based analysis of genes related to lead stress and their expression in the roots of Pogonatherum crinitum
Source: Front Plant Sci. 2022 Dec 14;13:1066329. doi: 10.3389/fpls.2022.1066329 (PMC9795032; doi:10.3389/fpls.2022.1066329)
Supplement: Supplementary file 1 [file DataSheet_1.docx]

Supplementary Material

# Supplementary Tables

SUPPLEMENTARY TABLE 1 Components of Hoagland nutrient solution

| **Nutrients/ Unit** | **Concentration** |
| --- | --- |
| KH_2_PO_4_ /(mg·L^-1^) | 0.680 |
| KNO_3_/(g·L^-1^) | 0.505 |
| MgSO_4_·7H_2_O/(g·L^-1^) | 0.492 |
| H_3_BO_3_/(g·L^-1^) | 0.002 |
| CuSO_4_·5H_2_O/(mg·L^-1^) | 0.079 |
| ZnSO_4_·7H_2_O/(mg·L^-1^) | 0.219 |
| MnCl_4_·H_2_O/(g·L^-1^) | 0.002 |
| (NH_4_)_2_MoO_4_/(mg·L^-1^) | 0.098 |
| Na_2_·EDTA/(mg·L^-1^) | 0.037 |
| FeSO_4_·7H_2_O/(mg·L^-1^) | 0.031 |
| Ca(NO_3_)_2·_4H_2_O/(g·L^-1^) | 1.180 |

SUPPLEMENTARY TABLE 2 Pb-tolerance-related genes in roots of *Pogonatherum crinitum* and primer sequences used in qRT-PCR

| **Gene ID** | **Primer Name** | **Primer sequences** | **Amplification product size** |
| --- | --- | --- | --- |
| GAPDH | Forward primer | GAAGCACAGTGACATCAGCC | 271 |
|  | Reverse primer | AAGATCCGAGGTGTACTTGTCC |  |
| CL17117.Contig11 | Forward primer | TCTTCCCCTTCTGGCCTTCT | 297 |
|  | Reverse primer | ATGCACAACACGTTCAGGGA |  |
| CL17117.Contig17 | Forward primer | ACATGGAGGGCTTTGGTGTC | 100 |
|  | Reverse primer | CATTTCACGCCACAAGTCGG |  |
| CL762.Contig2 | Forward primer | ATGGTTAAGGCTGTCTGCGT | 129 |
|  | Reverse primer | CTTGGCATTGGGTTCGTAGC |  |
| CL871.Contig1 | Forward primer | GCTGCTAGAGCCCATCAAGG | 85 |
|  | Reverse primer | TACCTCAACTGCCACGACTC |  |
| CL1174.Contig14 | Forward primer | GCACTATTGTCGGCGAGAGA | 156 |
|  | Reverse primer | ACGCAGCTTGAACAATACGC |  |
| Unigene7992 | Forward primer | GCAATATCCCTCAGCCCTGT | 176 |
|  | Reverse primer | ACCGTTGTATCTCCTTGGGC |  |
| CL12071.Contig | Forward primer | AGTGGGCCTCAGTGAACAAG | 241 |
|  | Reverse primer | GAAGTTCGCTTTGGTGGCTC |  |
| CL13989.Contig3 | Forward primer | TGTAAATGGCCCAGACACCG | 125 |
|  | Reverse primer | TGACCTTCCCGTTTTTGTCCA |  |
| CL5100.Contig1 | Forward primer | CCTCAGAACGCAGCAAGACT | 112 |
|  | Reverse primer | CGGTGCTCAGGATCAGTTCA |  |
| CL12532.Contig3 | Forward primer | GCTTCTCTTACAATGCGCCG | 233 |
|  | Reverse primer | CCATACGAACGCATCCCTGA |  |
| CL2332.Contig13 | Forward primer | CCGTTCTTCCTTGGCCAGTT | 239 |
|  | Reverse primer | TTTTTGCTTCCTCGTGCCCA |  |
| CL14444.Contig1 | Forward primer | GCAAAGAGGTTGGAGGGGAA | 116 |
|  | Reverse primer | GTCAGGCCAGCTTCCAAAAC |  |
| CL8121.Contig3 | Forward primer | AGACCTCAAACTGGATGGCG | 244 |
|  | Reverse primer | CCCATACCTGTGATGCTCCC |  |
| CL4795.Contig9 | Forward primer | TCAGGTCAAGAAGGCGATGC | 106 |
|  | Reverse primer | CAAGGTAGGCCAGGGAAACA |  |
| CL14340.Contig2 | Forward primer | ACGAGAGGTTGAGTCAGCAC | 193 |
|  | Reverse primer | CTTGGTAGTGATGGCTGGTGA |  |
| CL7623.Contig1 | Forward primer | TCTCCTGGTCCTGGTGCTAA | 271 |
|  | Reverse primer | TGCCACTAACTGCCCATCTG |  |
| CL1583.Contig2 | Forward primer | GCAACAAGAGAACTTCCCGC | 139 |
|  | Reverse primer | TTCCGGTGCAACTCAACAGA |  |
| Unigene46823 | Forward primer | TCAAGGAGCTGATGAGAGGC | 81 |
|  | Reverse primer | TGAGTGACAGAAATGGCCCG |  |

SUPPLEMENTARY TABLE 3 Analysis of transcriptome sequencing data in roots of *Pogonatherum crinitum*

| **Samples** | **RawData(bp)** | **CleanData(bp)** | **Q20(%)** | **Q30(%)** | **GC(%)** |
| --- | --- | --- | --- | --- | --- |
| CS1 | 45573892 | 42333758 | 97.59 | 93.59 | 53.14 |
| CS2 | 45573892 | 42354878 | 97.58 | 93.54 | 53.01 |
| CS3 | 45573892 | 42436374 | 97.53 | 93.41 | 52.80 |
| TS1 | 45573892 | 42311572 | 97.69 | 93.83 | 52.71 |
| TS2 | 47326734 | 43019658 | 97.72 | 93.98 | 53.12 |
| TS3 | 45573892 | 42250788 | 97.67 | 93.84 | 52.88 |

SUPPLEMENTARY TABLE 4 GO enrichment analysis of lead tolerance-related genes in the roots of *Pogonatherum crinitum*

| **GO term（Level 1）** | **GO ID（Level 2）** | **Function description** | **number of DEGs** |
| --- | --- | --- | --- |
| Biological Process | GO:0006355 | regulation of transcription | 918 |
|  | GO:0055085 | transmembrane transport | 612 |
|  | GO:0006952 | defense response | 390 |
|  | GO:0006979 | response to oxidative stress | 285 |
|  | GO:0016567 | protein ubiquitination | 263 |
|  | GO:0035556 | intracellular signal transduction | 244 |
|  | GO:0042744 | hydrogen peroxide catabolic process | 199 |
|  | GO:0055114 | oxidation-reduction process | 142 |
|  | GO:0009734 | auxin-activated signaling pathway | 138 |
|  | GO:0030001 | metal ion transport | 115 |
|  | GO:0006749 | glutathione metabolic process | 78 |
|  | GO:0032147 | activation of protein kinase activity | 77 |
|  | GO:0009755 | hormone-mediated signaling pathway | 74 |
|  | GO:0009611 | response to wounding | 71 |
|  | GO:0009738 | abscisic acid-activated signaling pathway | 61 |
|  | GO:0090630 | activation of GTPase activity | 50 |
|  | GO:0042542 | response to hydrogen peroxide | 37 |
|  | GO:0046274 | lignin catabolic process | 27 |
|  | GO:0006538 | glutamate catabolic process | 21 |
|  | GO:0006741 | NADP biosynthetic process | 19 |
|  | GO:0050665 | hydrogen peroxide biosynthetic process | 18 |
|  | GO:0032780 | regulation of ATPase activity | 5 |
| Cellular Component | GO:0005777 | Peroxisome | 166 |
|  | GO:0000307 | protein kinase holoenzyme complex | 64 |
| Molecular Function | GO:0004672 | protein kinase activity | 1700 |
|  | GO:0003700 | DNA-binding transcription factor activity | 759 |
|  | GO:0016301 | kinase activity | 504 |
|  | GO:0016705 | oxidoreductase activity, acting on paired donors, with incorporation or reduction of molecular oxygen | 321 |
|  | GO:0004842 | ubiquitin-protein transferase activity | 286 |
|  | GO:0004601 | peroxidase activity | 215 |
|  | GO:0016709 | oxidoreductase activity, acting on paired donors, with incorporation or reduction of molecular oxygen, NAD(P)H as one donor, and incorporation of one atom of oxygen | 138 |
|  | GO:0004364 | glutathione transferase activity | 101 |
|  | GO:0019901 | protein kinase binding | 86 |
|  | GO:0017137 | GTPase binding | 85 |
|  | GO:0015171 | amino acid transmembrane transporter activity | 75 |
|  | GO:0043295 | glutathione binding | 37 |
|  | GO:0052716 | hydroquinone:oxygen oxidoreductase activity | 27 |
|  | GO:0008106 | alcohol dehydrogenase (NADP+) activity | 17 |
|  | GO:0043783 | oxidoreductase activity, oxidizing metal ions with flavin as acceptor | 11 |
|  | GO:0016668 | oxidoreductase activity, acting on a sulfur group of donors, NAD(P) as acceptor | 8 |
|  | GO:0046915 | transition metal ion transmembrane transporter activity | 3 |
